# Mechanical Evaluation of PET-G 3D-Printed Wrist-Hand Orthosis: An Integrated Experimental and Numerical Approach

**DOI:** 10.3390/ma16186132

**Published:** 2023-09-09

**Authors:** Krzysztof Łukaszewski, Ratnesh Raj, Anna Karwasz

**Affiliations:** 1 Faculty of Mechanical Engineering, Poznan University of Technology, Piotrowo 3 STR, 61-138 Poznan, Poland; anna.karwasz@put.poznan.pl; 2 Department of Mechanical Engineering, Indian Institute of Technology (Indian School of Mines), Dhanbad 826004, Jharkhand, India; rtnsh07@gmail.com

**Keywords:** material extrusion, 3D printing, wrist-hand orthosis, modulus of elasticity, FEA simulation

## Abstract

Limb injuries frequently necessitate orthotic bracing, and the utilization of material extrusion (MEX) additive manufacturing (AM) or 3D printing offers a rapid and cost-effective means of producing orthoses. These characteristics are highly sought after in today’s orthotic market. The study focused on the mechanical strength analysis of the wrist-hand orthosis (WHO) made of PET-G filament. Experimental testing and simulation were employed to assess the properties of individualized wrist orthoses fabricated through the MEX AM process. Standard three-point bending samples were manufactured using PET-G filament on a low-cost MEX 3D printer, alongside orthotic fragments and complete orthosis. Experimental testing was performed using a universal testing machine, and results were juxtaposed with those from a finite element simulation model created in the Abaqus environment. This comprehensive research approach facilitates the comparison of the modulus of elasticity of the fabricated components, enabling a comparison between the mechanical properties of the complete wrist-hand orthosis (WHO) product and those of a conventional bending sample.

## 1. Introduction

The wrist-hand orthosis (WHO) serves as an external medical device designed to provide support to the wrist and hand during the treatment of upper limb conditions such as fractures, sprains, and injuries [1]. WHO devices offer stabilization, stiffness, and proper wrist positioning. They find application among both children and adults owing to their lightweight and breathable nature. These orthoses are constructed from materials such as textiles, Velcro, and plastics, facilitating breathability, moisture control, and comfort. Additionally, additive manufacturing (AM) presents a viable approach for their production [1,2]. This technology enables the creation of tailored orthotic solutions, precisely suited to an individual’s specific condition and requirements.

Utilizing a low-cost material extrusion (MEX) 3D printer, wrist-hand orthoses (WHO) were produced, circumventing the need for time-consuming and intricate tooling processes. This approach offers a heightened level of customization, enabling WHOs manufactured via 3D printing to be precisely tailored to individual patient requirements encompassing size, shape, and aesthetics. The efficient production timeline, coupled with a tailored fit for specific cases, contributes to heightened patient satisfaction and expedites the recovery process for injuries [3,4,5,6].

The primary objective of this research was to conduct a strength analysis on an orthosis crafted from polyethylene terephthalate glycol (PET-G) material utilizing a MEX 3D printer. The impetus behind performing the analyses detailed in the article also stems from the aspiration to enhance the product and comprehend the influence of production process parameters on the product. The goal was to identify the optimal process that aligns with the usage attributes of the object, encompassing factors such as weight, strength, and skin-friendliness.

The use of PET-G for the production of orthoses is beneficial due to [7]:

- being the most commonly recycled thermoplastic, it is not biodegradable but recyclable,
- having a very low thermal shrinkage,
- being odorless,
- not degrading in water or absorbing moisture, which is very important in case of contact with sweat,
- unlike PLA, not being brittle and being more flexible than PLA or ABS,
- being relatively easy to print (there are no requirements as to the type of 3D printer).

In their research, the authors also evaluate the use of other materials, as presented in papers [1] for ABS and [2] for PLA.

## 2. Literature Review

The material extrusion (MEX) process is an additive manufacturing (AM) process in which material, usually thermoplastic, is dispensed through a nozzle [8]. Additively fabricated items find diverse applications, notably within the medical field. Illustrative instances encompass surgical aids [9], implants including cochlear implants [10], and personalized prosthetics and orthotics such as the WHO [11]. Objects fabricated through the MEX technique are produced by layering plasticized thermoplastic material [12] in the form of filaments. Frequently employed materials encompass PET-G, polylactic acid (PLA), acrylonitrile butadiene styrene (ABS), high-impact polystyrene (HIPS), polyvinyl alcohol (PVA), polyethylene terephthalate (PET), and thermoplastic polyurethane (TPU) thermoplastics; however, the assortment of materials and applications continue to expand [13].

Certainly, embedding sensors within structures is indeed one of the noteworthy advantages that MEX offers. This capability allows for the creation of smart structures, enabling enhanced monitoring and functionality [12,14].

The MEX 3D printing is distinguished by pronounced anisotropy in its strength. The processing conditions wield substantial influence over the mechanical strength of the fabricated parts. Even a slight adjustment in a single process parameter, such as altering the orientation of the product within the workspace of the 3D printer, can yield noteworthy disparities in strength of the manufactured samples [15]. In paper [16], the authors demonstrate that despite the additive layer-by-layer formation of parts, determining product properties analytically necessitates an approach distinct from the existing presumed models for laminated composites. The research carried out by the authors in [17] proposes the analysis of products produced using the MEX method as orthotropic composites, wherein the extruded material fibers constitute the matrix and the fillers represent voids. Comparing two simulation models created with Abaqus software affirms that treating the MEX-produced item as an orthotropic anisotropic material is appropriate. Leveraging the capabilities of the Abaqus system, calculations for anisotropic materials become feasible, enabling the incorporation of a suitable modulus of elasticity values for specific orientations [18]. Both solid and layered models yielded outcomes akin to those acquired through testing tangible samples [19]. A more complex endeavor involves formulating an analytical model for a multi-material WHO crafted within a single MEX process through dual extruders [20].

An additional significant consideration, indirectly entwined with various technical parameters and the geometry of the product in the context of properties linked to items produced via the MEX method, pertains to the temperature status of the preceding layer when applying the subsequent layer. As elucidated by the authors of [21], the temperature of the underlying layer plays a pivotal role in determining the ultimate tensile strength (UTS) value. This temperature parameter is contingent upon multiple factors, encompassing bed and nozzle temperatures, as well as printing speed. In instances involving smaller-sized samples, material extruded in the initial layer has limited time to cool in comparison to products with larger dimensions. Consequently, it is plausible to assume that a similar correlation might manifest in the context of other strength characterization metrics for products generated through the MEX method.

The investigation conducted in [21] further establishes that extrusion efficiency profoundly affects strength parameters, specifically due to the presence of volumetric errors inherent to the nature of the MEX process. These errors correspond to regions where no material is deposited, subsequently influencing the overall density of the final product. Although a simplistic viewpoint might posit that products of similar density would exhibit analogous properties, comprehending the occurrence and location of such volumetric errors within a specific product holds significance. Nonetheless, by gauging the mass of geometrically identical samples from a designated series, it becomes possible to deduce the reproducibility of the manufacturing process.

MEX 3D printing offers the advantage of swiftly producing fully individualized, unitary items, a notable contrast to conventional manufacturing methods such as injection molding. Nevertheless, fabricating prototypes to conduct essential experiments for refining novel designs remains a laborious and financially impractical endeavor. As such, several researchers have endeavored to employ computational techniques to anticipate a product’s mechanical responses [19]. Consequently, this approach substantially curtails the necessity for extensive experimental measurements, leading to cost and time reductions in the manufacturing process, ultimately expediting the product’s journey to the consumer [22]. This acceleration can prove pivotal, particularly in medical applications where swifter delivery holds paramount importance, particularly for tailor-made products designated for specific individuals.

It is important to highlight the absence of comprehensive methodologies within the existing literature that sufficiently detail the acquisition of material data for items produced through 3D-printing technology, particularly for the purpose of numerical testing, particularly within the domain of medical device applications. A case in point can be found in the literature [23], which introduces a methodology for conducting simulations and experimental analyses on an open-wrist orthosis (comprising solely the lower part of the shell). Notably, the applied force was exerted from the distal aspect, i.e., the palm side. However, the discussion in this paper omits any mention of the material data employed in the simulations. Numerous papers that elucidate simulation tests utilizing the finite element method fail to establish a comparison between outcomes attained through experimental tests. This includes papers that exclusively employ mechanical parameters of the material [24,25,26,27] and those that solely delineate the technique for applying loads to a simulated model of the orthosis [28].

The review of the existing literature underscores the rationale for conducting a study with the objective of ascertaining the applicability of a numerical approach in characterizing products engendered through MEX 3D printing, specifically in the context of wrist orthoses. These orthoses, employed in post-traumatic treatment, necessitate prompt delivery to patients. Consequently, the numerical model must satisfy two pivotal criteria. Firstly, it must yield realistic outcomes that facilitate orthosis geometry design and rapid manufacturing using the MEX technique. Secondly, the computations must be expedient, enabling the immediate commencement of individual orthotic production subsequent to the acquisition of anthropometric data from the patient.

The innovation behind crafting hand orthosis using PET-G via 3D printing stems from its revolutionary approach to producing personalized orthotic devices. This method encompasses several distinct elements: Firstly, the selection of PET-G material for 3D printing hand orthoses introduces a contemporary and pioneering choice. PET-G is renowned for its robustness, flexibility, and suitability in medical contexts, rendering it an exceptional candidate for constructing orthotic devices that ensure both hand support and comfort. The utilization of 3D-printing technology for shaping hand orthoses heralds a transformative shift in the manufacturing landscape. This technique facilitates intricate designs and bespoke fittings that prove challenging to achieve using conventional methods. It empowers the generation of intricate geometries meticulously tailored to each individual’s distinct hand structure. The hallmark of this novelty rests in the capability to customize hand orthoses according to the precise requirements of each patient. Unlike mass-produced counterparts, this approach guarantees an exact fit and heightened functionality, fostering patient comfort and adherence to prescribed treatment protocols. The incorporation of simulation studies in this research introduces an inventive dimension. By harnessing numerical simulations, the study strives to anticipate the behavior and performance of the produced hand orthosis across diverse scenarios. This strategy optimizes the testing procedure and notably truncates the time traditionally consumed by physical experimentation. Further innovation manifests through the simulation-driven design approach, allowing for rapid design iteration and enhancement. Researchers can make well-informed decisions about design parameters, material thicknesses, and structural components by virtually assessing their influence on the orthosis’ efficacy. This results in a more refined and efficacious final product. The amalgamation of PET-G 3D-printed hand orthoses and simulation-driven design carries considerable relevance within the realm of medical devices. This amalgam has the potential to expedite the development of bespoke solutions for patients, particularly in post-traumatic scenarios where prompt intervention plays a pivotal role in recovery.

In summary, the pioneering nature of hand orthosis fabrication using PET-G via 3D printing arises from its utilization of state-of-the-art materials, personalized customization, and simulation-guided design. This approach not only elevates patient outcomes but also signifies a significant advancement in the arena of orthotic device manufacturing and patient care.

## 3. Materials and Methods

### 3.1. Research Plan

The orthotic must have the best possible strength properties while being as light as possible to allow for comfortable use. In addition, the manufacturing time for the orthotic must be minimized so that the finished product is available within hours of the patient’s limb being scanned. To achieve this goal, a series of experiments and simulation trials were planned.

The following activities were conducted:

- sample preparation: standard 3-point bending samples, samples of the orthotic center without and with openings, and samples of the entire orthotic shape,
- measuring the density of the samples,
- bending test,
- modeling and simulation (FEM) of sample loads,
- comparison of displacement values from experiment and simulation, and determination of measured elastic modulus based on simulation results.

Given the equivalence of the force employed in the simulation (Ps) to the force derived from experimentation (Pe), along with matching mass and geometric attributes between the simulation and experiment, Equation (1) allows for the direct determination of the actual modulus of elasticity (*E_e_*).

(1) $$E_{e}=\frac{U_{is}\cdot E_{s}}{U_{ie}}$$

where:

*E_e_*—real value of elastic modulus [MPa],
*E_s_*—simulated value of elastic modulus [MPa],
*U_ie_*—experimentally obtained displacement value [mm],
*U_is_*—simulated displacement value [mm].

The methodology and results of the work performed are detailed later in the article.

### 3.2. Manufacturing of Samples

A FlashForge (Jinhua, China) Creator Pro 3D printer was used in the tests. The values of the most important operating parameters of the manufacturing process are listed in Table 1.

PET-G material was used in the production of all samples. All samples were made from one batch of material from the same manufacturer.

Product data, one spool of PET-G filament, trade name “PET-G Standard 1.75 mm Blue Sky Transparent 0.8 kg”. Manufacturer, responsible entity: ROSA PLAST SP. z o.o.ul. Hipolitowska 102B, 05-074 Hipolitów, Poland. Recommended working parameters for 3D printing [29]:

- extrusion temperature 220–250 [°C].

Physical parameters [30]:

- density 1.29 [g/cm^3^],
- heat deflection temperature (HDT) 78 [°C].

**Figure 1:**
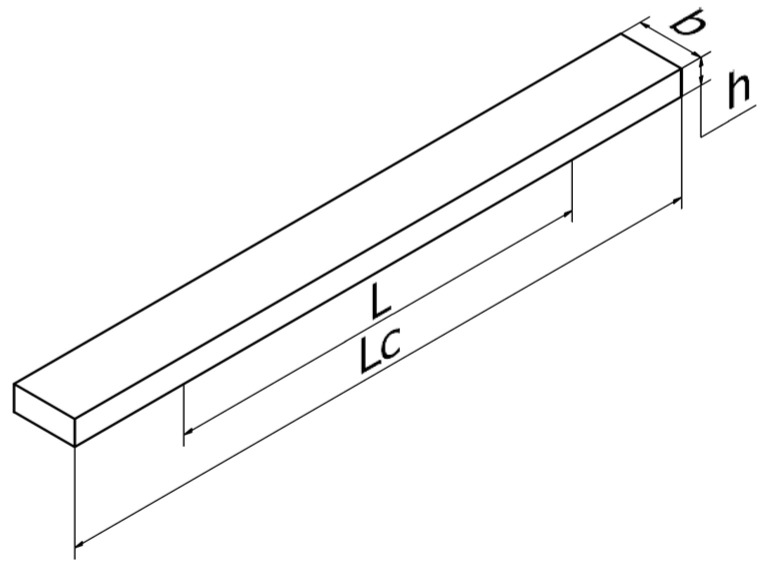
Dimensions of the test sample—iso view with the marked spacing of the supports of the testing machine: Lc = 100 mm, L = 64 mm, h = 4 mm, b = 10 mm.

In the first step, a standard test sample (P) was prepared for the 3-point bend test (Figure 1). This test sample was prepared according to the guidelines of EN ISO 178 [30]. Next, a sample in the shape of the central part of the brace (MP) was prepared. The third type was an orthosis-shaped sample (F), which was not divided into two shells. The last type (fourth type) of test sample was an orthotic-shaped sample consisting of a lower shell (SB) and an upper shell (ST).

Straight infilling (honeycomb) of 10% was used for all samples. The outer contour consisted of two contours 0.3 mm wide.

**Figure 2:**
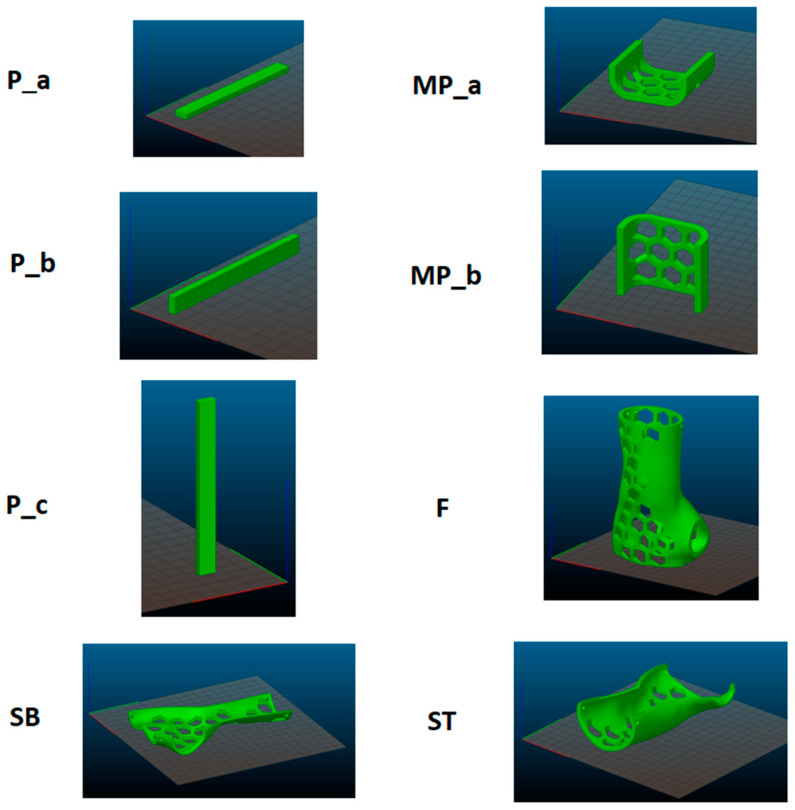
Designation of a series of samples and their arrangement in the working chamber.

The 3D printer produced a series of samples in different orientations in the working chamber (see Figure 2). Refer to Figure 2 for P-5 samples, MP-3 samples, P samples, ST samples, and SB-2 samples.

**Figure 3:**
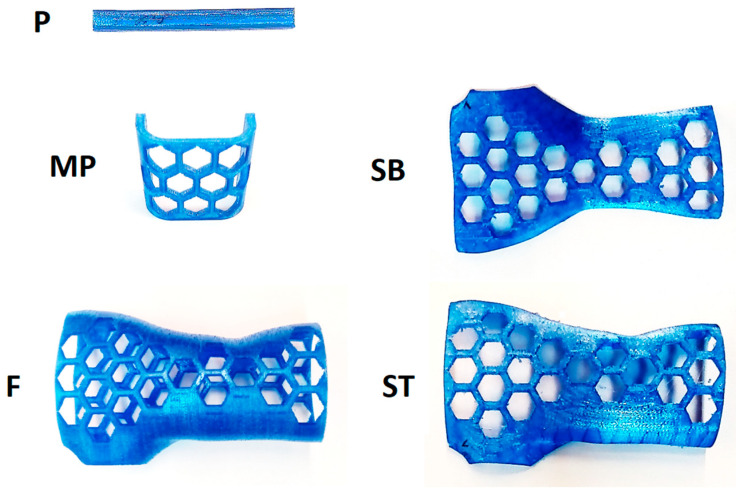
Manufactured samples, series: P, MP, SB, F, ST.

Figure 3 shows the original fabricated samples. Every produced sample underwent a meticulous visual assessment to identify any structural irregularities resulting from potential manufacturing errors. Consequently, no defects were identified that would render the samples unsuitable for continued utilization in the study.

### 3.3. Methodology of Bending Tests

**Figure 4:**
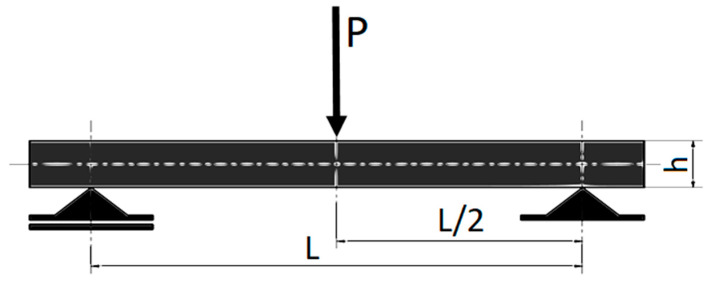
Scheme of the three-point bending test: P—bending force, L—distance between supports, h—sample height, based on [30].

Strength tests of the specimens were performed using the universal testing machine WDW-5D-HS. The PN-EN ISO 178 standard for three-point bending tests was used, where cubic specimens with a ratio of height h to strut spacing L of L/h = 16 and 64/4 = 16 was taken for the tests. Figure 4 shows the schematic representation of the three-point test of fabricated standard samples [30].

**Figure 5:**
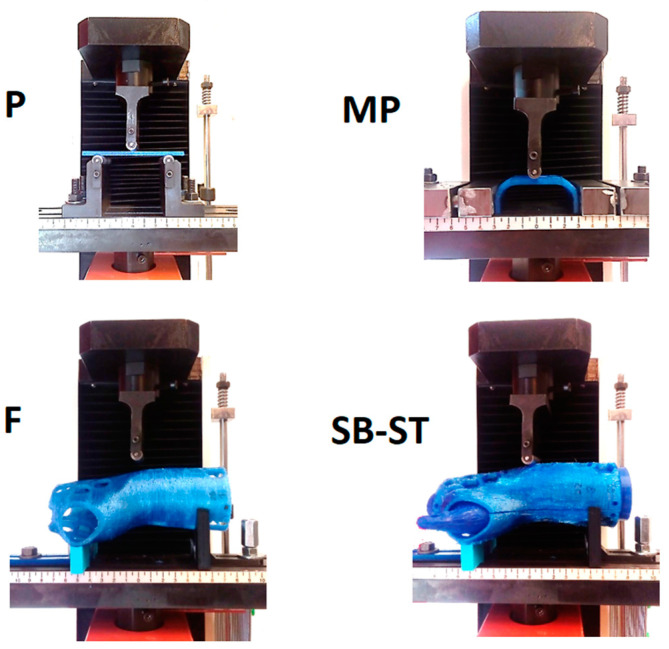
Three-point bending of the sample—view of the working tooling of the testing machine, bending of all types of samples.

Figure 5 shows a photograph of the sample during the bending test. The operating mechanism of the grips of the testing machine for the orthosis and its fragments was fabricated using 3D printing, and the main criterion of suitability of these was to ensure the stability of the selected sample during the measurements.

### 3.4. Methodology of Finite Element Analysis

The Abaqus 6.12, Standard module (statics), was used for the simulation tests. To obtain correct results in the modeling, higher-order spatial finite elements with a central node between vertex nodes were used. This is because with finite element meshes, displacement values are computed at each node, so the more nodes, the more correct the values obtained. The results of the two finite element meshes overlaid on the sample model were compared with the displacement (Ui) results obtained from the experimental tests. A triangular element with 10 nodes on the element (C3D10) was used to apply the meshes.

**Figure 6:**
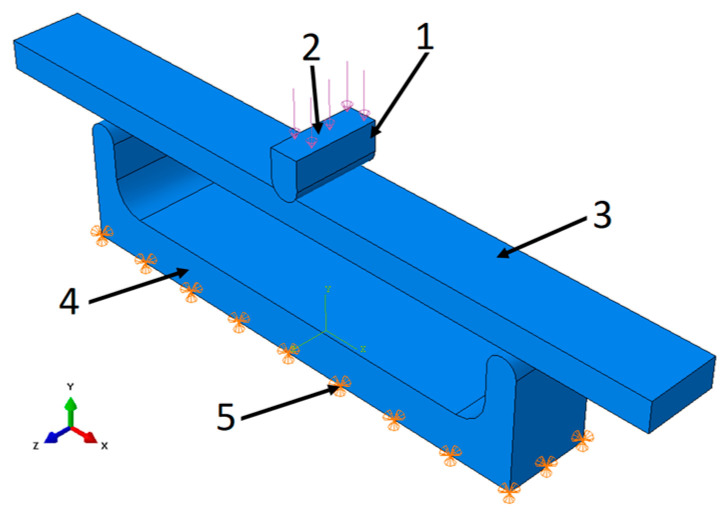
Simulation model of P series samples: 1—pressing element, 2—place of application, 3—tested sample, 4—base, 5—place of receiving force.

The simulation model for the basic standard sample was created in the form of an assembly that fully reproduced the operating elements of the testing machine (Figure 6).

**Figure 7:**
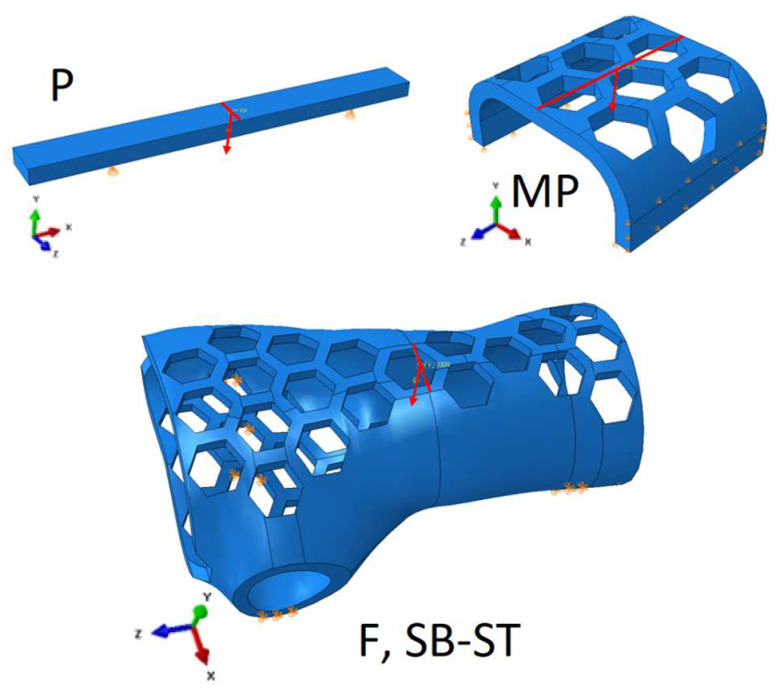
Boundary conditions and loading method for standard test sample (P), central part of the brace (MP), orthosis-shaped sample (F), and orthotic-shaped sample consisting of a lower shell and upper shell (SB-ST).

To confirm the possibility of reducing computation time, the authors also applied direct loading to the base sample using forces and boundary conditions. The results showed that the difference between the results obtained with indirect loading using the working elements and those obtained with direct loading was less than 1%, and the computation time was reduced by a factor of 3, from 3 min to 1 min. For the remaining specimens, the tester’s working elements were not modeled and were limited to applying the appropriate forces and boundary conditions for the specimen (Figure 7). Modeling with work elements requires the use of contact surfaces, which significantly increases computation time compared to applying static loading to the specimen by applying forces through stationary manipulation of the work tool or the assembly table (holder) of the testing machine. Calculation time is important in terms of its potential use in production systems for product mass customization.

**Figure 8:**
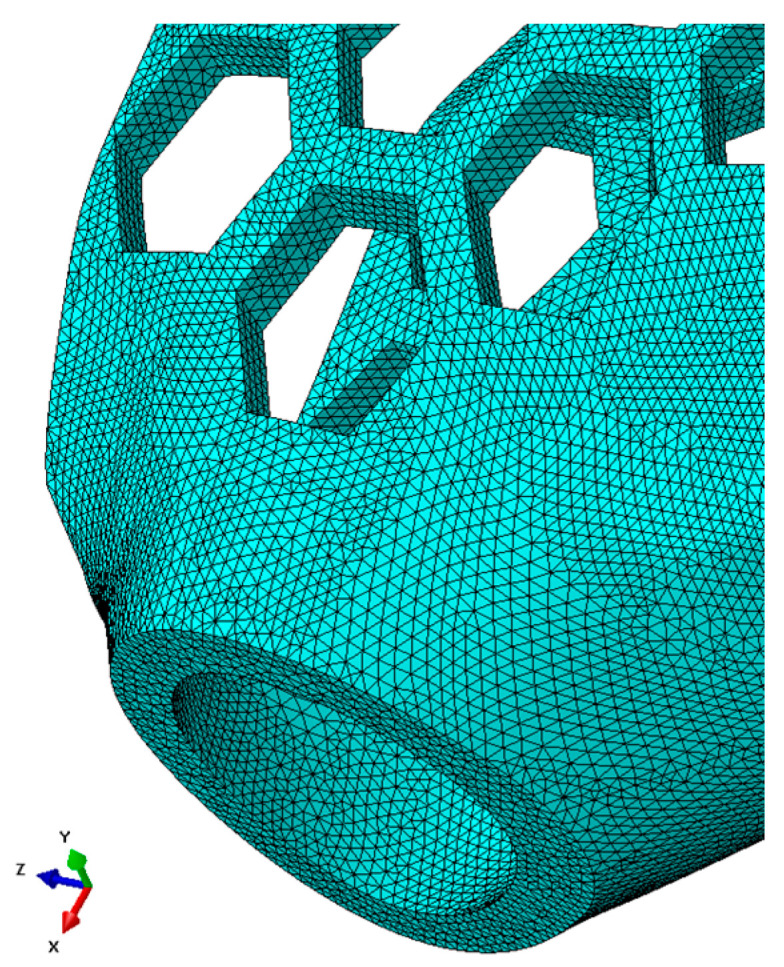
Finite element mesh superimposed on the test specimen in the shape of the entire orthosis of high geometric complexity (partial view).

A diagram of a finite element mesh fragment of the orthotic model that accurately reflects the shape of the curvature of the orthotic is shown in Figure 8. The mesh parameters for all types of samples are shown in Table 2.

For ease of computation, all types of samples were given the same Young’s modulus E = 1000 MPa and Poisson number = 0.38.

## 4. Results

### 4.1. Bending Test Results

**Figure 9:**
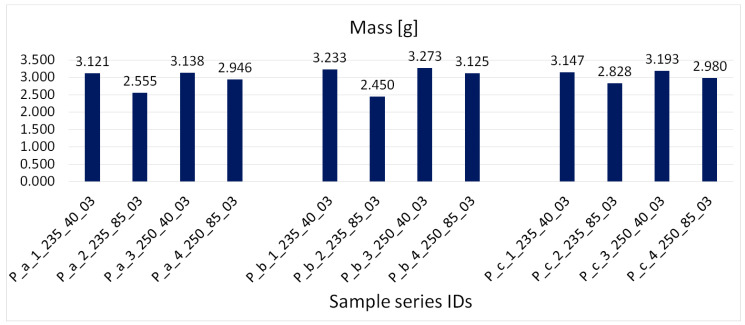
The obtained average mass values of the primary samples, samples ordered according to orientation in the working chamber.

**Figure 10:**
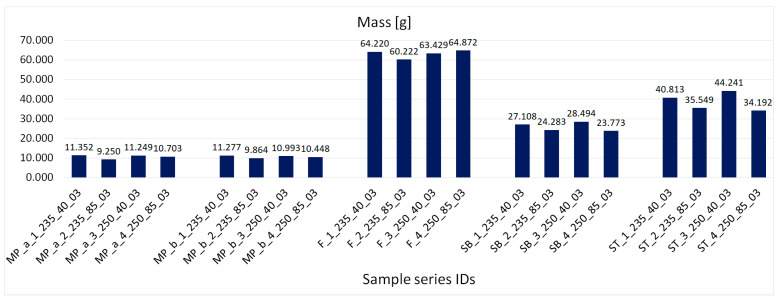
Obtained average mass values of samples with complex geometry, samples ordered according to orientation in the working chamber.

**Figure 11:**
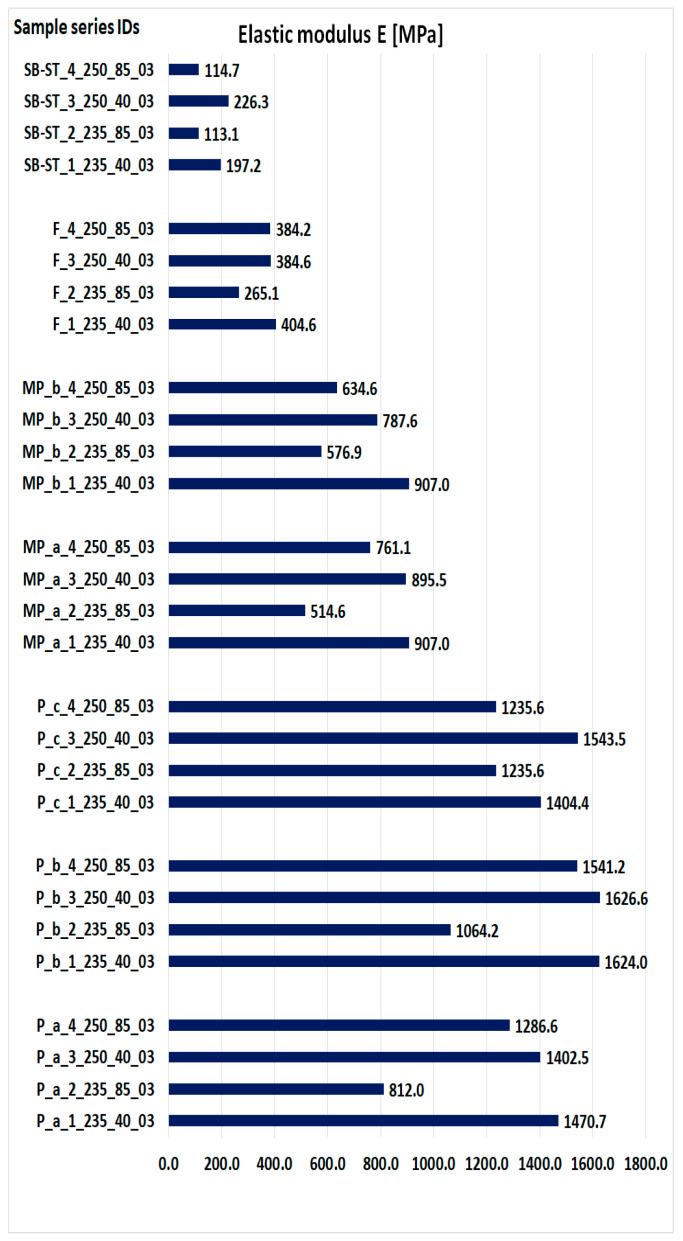
Obtained average density values, samples ordered according to the manufacturing process.

Figure 9 shows the obtained average mass values of primary samples ordered according to their orientation in the working chamber. Figure 10 shows the obtained average mass values of samples with complex geometry, with samples ordered according to orientation in the working chamber. Figure 11 shows the obtained average density values, with samples ordered according to the manufacturing process.

### 4.2. Bending Test Results

**Figure 12:**
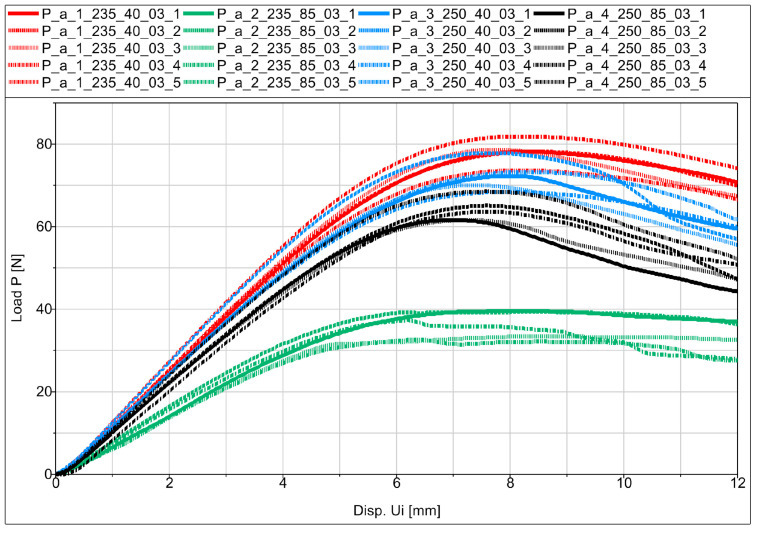
Force–displacement relationship diagram for samples from the P_a series.

Experimental flexural testing of the manufactured specimens allowed us to obtain force (P)–displacement (Ui) graphs for the individual specimens in the series. Figure 12 shows a schematic diagram of the basic sample P_a in the series.

**Figure 13:**
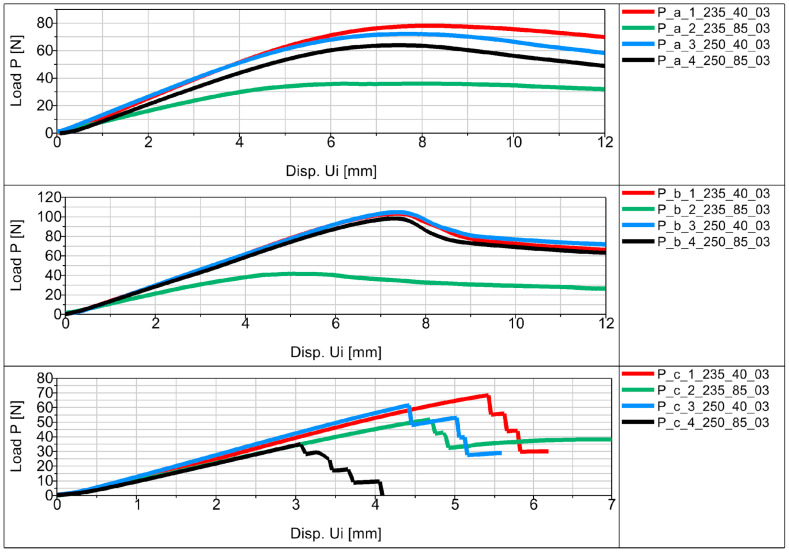
Force (P)–displacement (Ui) dependence from average values for basic samples.

**Figure 14:**
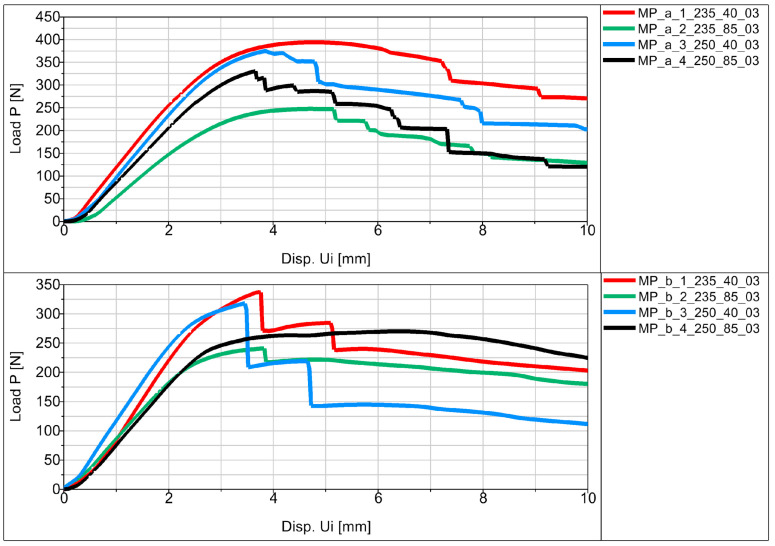
Force (P)–displacement (Ui) dependence from average values for samples in the shape of the middle part of the orthosis.

**Figure 15:**
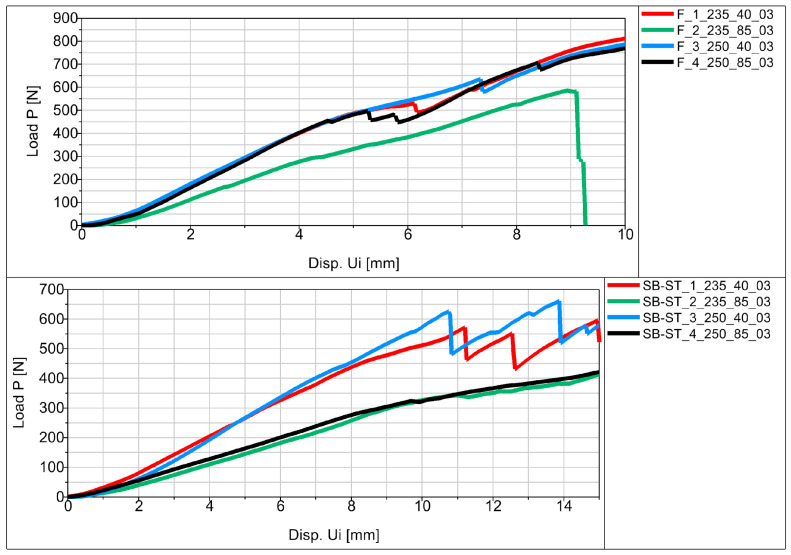
Force (P)–displacement (Ui) dependence from average values for samples in the shape of the entire orthosis.

The average values of displacement for all samples are shown in Figure 13, Figure 14 and Figure 15 in the form of force (P)–displacement (Ui) diagrams.

Analyzing the obtained figures with averaged values (Figure 13, Figure 14 and Figure 15), it should be noted that perfect linearity is not obtained due to the internal structure of the thermoplastic, i.e., the overlapping of the individual layers of the thermoplastic. The approximate linearity obtained must be combined with temperature variations associated with the position of the nozzle applying the material and variable heat dissipation conditions that affect the bonding forces at the boundaries between the layers and the path.

### 4.3. Finite Element Analysis Results

A comparison of vertical displacement values obtained from simulation tests using the FEM method (Ui = U2 = Uy) is shown in Table 3.

The values of displacement Ui shown in Table 3 are for reference only and were used to determine the actual modulus values.

### 4.4. Elastic Modulus of Samples

**Figure 16:**
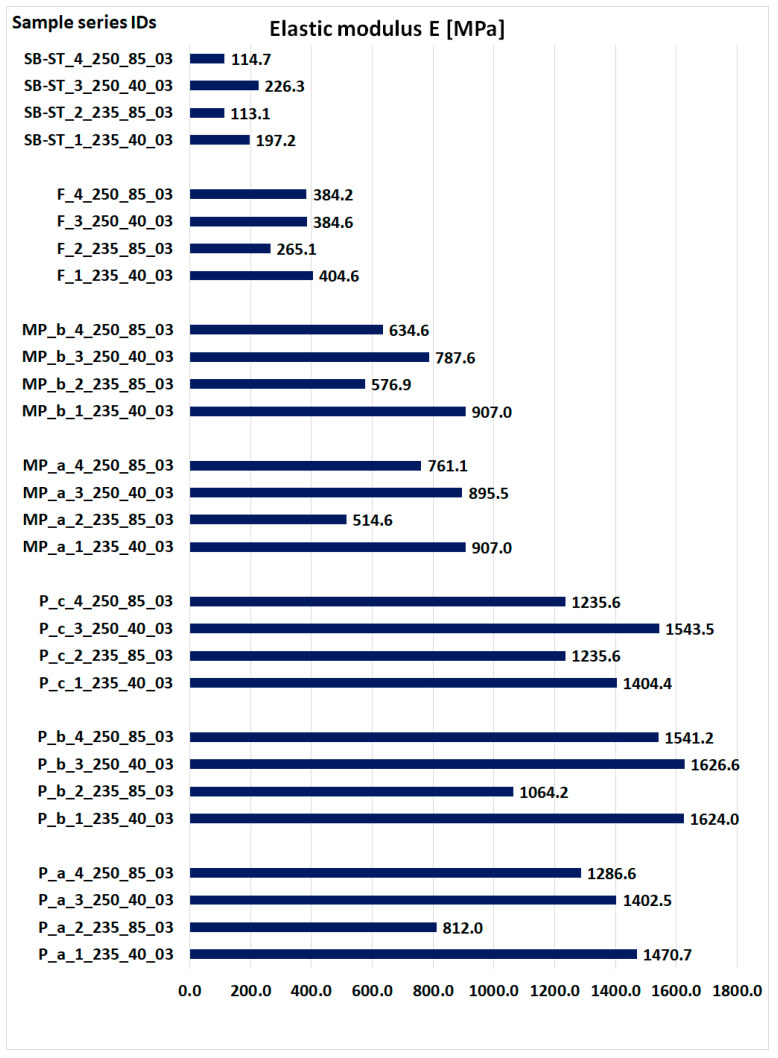
The obtained values of the modulus of elasticity.

Figure 16 presents the obtained values of the modulus of elasticity for all types of samples. The values were determined on the basis of the results obtained on real objects during three-point bending and the values of displacements obtained by simulation, substituted in Equation (1).

### 4.5. Result Comparison

Comparable values for density, modulus of elasticity, force, and displacement are tabulated below (Table 4).

Sample SB-ST_3_250_40_03 with low speed and higher temperature shows better results, and thus we obtain better mechanical properties, which results in the highest strength. The same was obtained for samples of the same orientation in a working chamber of a different shape (series of samples Pa, Pb, MP_a).

## 5. Discussion

### 5.1. Effect of Printing Parameter Variations in Weight and Density

Different combinations of temperature and printing speed were used to 3D print all the samples. The standard bending samples were printed in three orientations, while samples in the shape of the central part of the orthosis (MP) was printed in two orientations, zero and 90 degrees. The WHO was printed in the full 90-degree orientation (F), and two split sections, SB and ST, of the WHO were printed in the zero-degree orientation. The weight and density of the printed objects were examined.

The observations recorded in Figure 9, Figure 10 and Figure 11 indicated that higher weight and density were associated with higher extrusion temperatures. Increased temperature improved the material’s flowability, resulting in more material being extruded and deposited, leading to higher weight and density. Conversely, it was found that weight and density decreased with increasing extrusion speed. Higher speeds caused the nozzle to move faster between locations, resulting in inadequate material extrusion at certain points. However, at a speed of 40 mm/s, there was enough time for proper material deposition on the print bed. When considering all the variations together, it was concluded that at an extrusion speed of 40 mm/s, temperature variations had a minimal impact on the density and weight of the printed products. However, at a speed of 85 mm/s, the influence of high temperature became more noticeable. At high temperatures, the weight was approximately comparable to that of products printed at 40 mm/s, while the weight was significantly lower at a temperature of 235 °C. This confirms the assertion that higher temperature and extrusion result in increased weight, as supported by density and weight measurements.

A slight discrepancy was observed in the printed SB and ST samples (Figure 10 and Figure 11). At a speed of 40 mm/s, there was a noticeable difference in weight due to temperature variation. However, at 85 mm/s, the weight difference was almost the same regardless of temperature variation. This could be attributed to the zero-degree printing orientation and the extensive nozzle movement. At 40 mm/s, the slower nozzle movement allowed enough time for the deposited material to solidify properly, preventing intermixing or overlapping as specified by the slicing instructions. Therefore, higher weight was achieved at higher temperatures due to improved flowability. Conversely, at 85 mm/s, the lack of weight difference can be attributed to the shorter nozzle movement time between locations, which was insufficient for the proper solidification of previously deposited materials. These results demonstrate an inverse relationship compared to previous samples with smaller build areas.

### 5.2. Effect of Printing Parameter Variations on Strength

All samples showed similar load-bearing capabilities when printed at a speed of 40 mm/s, regardless of the extrusion temperatures of 250 °C and 235 °C shown in Figure 13, Figure 14 and Figure 15. However, at a speed of 85 mm/s, there was a notable difference in strength. Samples printed at 250 °C exhibited higher strength, while those printed at 235 °C had significantly lower strength. These findings suggest that a 40 mm/s extrusion speed is more effective for producing parts with good load-bearing capabilities. On the other hand, 85 mm/s is not as effective, except when combined with higher extrusion temperatures (250 °C), which yielded comparable strength to samples printed at 40 mm/s. One exception was observed in the printed SB-ST samples (Figure 15), which consist of a phantom inside and split parts of the WHO. The strength of samples printed at 85 mm/s was lower than those printed at 40 mm/s, and both were nearly equal.

Fracture stages were observed in various samples, including P_c, MP_a, MP_b, F, and SB-ST (Figure 13, Figure 14 and Figure 15). In the standard bending samples of P_c, the fracture stages occurred due to the vertical printing orientation (90 degrees with respect to the bed), which weakened the layer joinings parallel to the loading arm of bending. Fracture stages represented multiple fractures occurring at different layer joinings. In contrast, samples P_a and P_b had vertical and horizontal layer placements, resulting in perpendicular joinings to the loading arm, thus exhibiting higher strength and no stages in between. Similar fracture stages were observed in the MP samples, with shallower stages in MP_a and deeper stages in MP_b, indicating multiple fractures. The steeper stages in MP_b were due to the vertical upward printing orientation, resulting in 0.3 mm layers perpendicular to the loading arm compared to MP_a, which had whole-area layers facing the bending roller. In the vertically printed WHO samples at 40 mm/s extrusion speed, two fracture stages were observed, with the sample printed at 250 °C experiencing a delayed first fracture compared to the sample printed at 235 °C. This delay could be attributed to the higher temperature, leading to better deposition, proper overlapping, and adequate adhesion between layers.

In the testing results of SB-ST, three fracture stages were visible in samples printed at 40 mm/s, while no multiple stages were observed in samples printed at 85 mm/s. Insufficient material deposition and the presence of pores between layers at 85 mm/s weakened the entire sample, resulting in consistent weak strength throughout, similar to the weak corners of the printed honeycomb feature. Among the standard bending samples, P_b exhibited the highest load-bearing capability compared to P_a and P_c. This outcome was influenced by the printing orientation, as the vertical upward orientation weakened P_c due to weaker layer joinings parallel to the loading arm. Layer joinings and adhesion are generally weaker sections in additive manufacturing samples, leading to abrupt failure and reduced strength.

MP_a demonstrated higher strength and load-bearing capabilities up to greater displacement due to its selected printing orientation with the whole-area layer facing the bending roller. This success inspired the printing of the WHO parallel to the bed, resulting in the printing of split sections SB and ST. The vertically printed WHO without splits (F) did not allow for the insertion of a phantom during testing. SB-ST, which is the WHO tested with a phantom inside, exhibited three different fracture stages. The hand phantom consisted of an outer elastic layer imitating soft tissues, skin and muscles, and a hard core imitating bone. Both F and SB-ST were tested for bending, and F demonstrated good strength even without any support or phantom inside. This could be attributed to its single-run manufacturing. However, the presence of gaps between split sections, between the shell of SB and ST, and the phantom led to a decrease in strength. Nevertheless, the results up to the first fracture stage of SB-ST were nearly equal to those of F. The support provided by the phantom during SB-ST testing resulted in higher displacement extensions compared to the F samples. Overall, the split WHO is recommended for its utility, ease of fixing and replacing, and higher extensional displacement capabilities.

The elastic modulus measures a material’s stiffness, which directly impacts its strength. A higher modulus indicates less elastic deformation, signifying higher stiffness. Among the standard bending samples, P_b exhibited the highest elastic modulus (Figure 16). Notably, the P_c samples printed at 235 °C and 85 mm/s showed higher modulus values than the other two samples printed under the same parameters. Additionally, the P_c samples printed at 250 °C and 40 mm/s exhibited higher strength than the P_a samples printed under the same parameters. In the MP samples, MP_a had a higher modulus for samples printed at 250 °C, while MP_b had a higher modulus for samples printed at 235 °C and 85 mm/s. When printed at 235 °C and 40 mm/s, both MP samples had almost equal modulus values. Comparatively, the F sample had higher modulus values than SB-ST, indicating higher stiffness. The modulus of SB-ST was approximately half that of F.

## 6. Conclusions

The most significant factor influencing the enhancement of strength properties is the extrusion speed. A lower extrusion speed ensures precise replication of object contours, preventing the formation of weakening voids. The tested speeds were 40 [mm/s] and 85 [mm/s].

In comparison, temperature’s impact on object strength during the manufacturing process is relatively less pronounced. Results at temperatures of 235 °C and 250 °C do not distinctly establish whether higher temperatures correlate with improved strength. The temperature range of 220 to 250 °C aligns with the manufacturer’s recommendations for used filament. Combinations utilizing an extrusion speed of 40 [mm/s] yielded the most favorable outcomes with both variants of opted temperatures (235 and 250 °C). A well-optimized selection of process parameters ensures the product attains suitable properties. This approach is employed for evaluating the mechanical attributes of WHOs. The study’s findings underscore that the proposed method is viable for assessing the strength of personalized orthotics during design, considering printing parameters such as extrusion speed, temperature, and orientation.

The computational time required for these numerical calculations using a dedicated unit is just a fraction of the actual production time. Moreover, precise customization of product geometry for each patient might lead to reduced manufacturing time, as it eliminates the need for excess material extrusion. Essentially, incorporating numerical calculations could expedite the delivery of personalized orthotics to patients. Consequently, exploring the integration of a numerical simulation module into the product mass customization system is a justifiable avenue for further investigation.

As the incremental manufacturing process with MEX 3D printing hinges on numerous variables, additional research is warranted to expand the simulation library with fresh material data. Further, it is advisable to validate simulations for alternative thermoplastic materials and for the entire orthosis geometry using real models.

## Figures and Tables

**Table 1:** Main parameters of 3D-printing process.

Sample No.	Extrusion Temperature [°C]	Extrusion Speed [mm/s]	Layer Thickness [mm]	Machine	Material
1	235	40	0.3	FlashForge Creator Pro	PET-G
2	235	85			
3	250	40			
4	250	85			

**Table 2:** Parameters of the applied finite element mesh on the test samples.

Series Designation	Shape of Element	Type	Global Size	Amount of Nodes	Amount of Elements
P	Triangular	C3D10	1 mm	42,517	27,456
MP	Triangular	C3D10	1 mm	158,610	102,752
F, SB-ST	Triangular	C3D10	1 mm	856,431	561,274

**Table 3:** U2 vertical displacement for all sample series.

Series	Force Value *p* Displacement Ui	Result as a Colorful Map
P	P = 10 [N] Ui = 1.028 [mm]	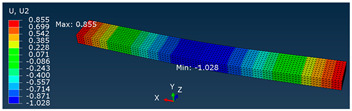
MP	P = 100 [N] Ui = 0.634 [mm]	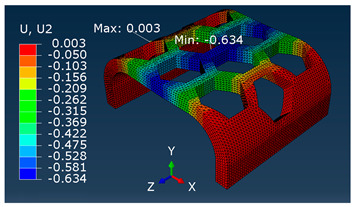
F, SB-ST	P = 100 [N] Ui = 0.320 [mm]	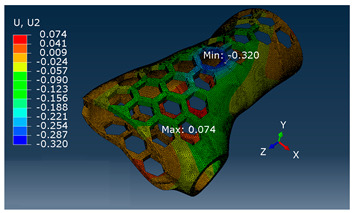

**Table 4:** Cumulative results of the maximum values obtained for a given series of samples.

Series Designation	Density [g/cm^3^]	Obtained E Modulus [MP]	The Maximum Force Obtained from the Average P [N]	Displacement Value from Maximum Force Ui [mm]
P_a_1_235_40_03	0.780	1470.7	78.04	8.25
P_a_2_235_85_03	0.639	812.0	36.00	6.31
P_a_3_250_40_03	0.785	1402.5	72.16	7.79
P_a_4_250_85_03	0.736	1286.6	63.96	7.59
P_b_1_235_40_03	0.808	1624.0	103.16	7.39
P_b_2_235_85_03	0.613	1064.2	41.56	5.11
P_b_3_250_40_03	0.818	1626.6	104.76	7.35
P_b_4_250_85_03	0.781	1541.2	98.32	7.29
P_c_1_235_40_03	0.787	1404.4	68.44	5.42
P_c_2_235_85_03	0.707	1235.6	51.80	4.69
P_c_3_250_40_03	0.798	1543.5	61.60	4.24
P_c_4_250_85_03	0.745	1235.6	35.00	3.06
MP_a_1_235_40_03	0.758	907.0	394.20	4.80
MP_a_2_235_85_03	0.618	514.6	247.40	4.83
MP_a_3_250_40_03	0.751	895.5	374.60	3.86
MP_a_4_250_85_03	0.715	761.1	330.87	3.66
MP_b_1_235_40_03	0.753	907.0	337.00	3.73
MP_b_2_235_85_03	0.659	576.9	241.133	3.83
MP_b_3_250_40_03	0.734	787.6	318.27	3.46
MP_b_4_250_85_03	0.698	634.6	270.4	3.57
F_1_235_40_03	0.786	404.6	829.40	10.68
F_2_235_85_03	0.737	265.1	882.50	10.95
F_3_250_40_03	0.777	384.6	585.60	8.94
F_4_250_85_03	0.794	384.2	932.60	11.79
SB-ST_1_235_40_03	0.862	197.2	649.10	17.79
SB-ST_2_235_85_03	0.772	113.1	605.20	22.09
SB-ST_3_250_40_03	0.906	226.3	741.30	17.45
SB-ST_4_250_85_03	0.756	114.7	588.00	22.64

## Data Availability

Not applicable.

## References

[1] Łukaszewski K., Wichniarek R., Górski F. (2020). Determination of the Elasticity Modulus of Additively Manufactured Wrist Hand Orthoses. Materials.

[2] Raj R., Dixit A.R., Łukaszewski K., Wichniarek R., Rybarczyk J., Kuczko W., Górski F. (2022). Numerical and Experimental Mechanical Analysis of Additively Manufactured Ankle–Foot Orthoses. Materials.

[3] Štefanovič B., Danko M., Michalíková M., Bednarčíková L., Rajťúková V., Tóth T., Trebuňová M., Hudák R., Živčák J. (2021). Orthoses Development Using Modern Technologies. Prosthetics and Orthotics.

[4] Liu Z., Zhang P., Yan M., Xie Y., Huang G. (2019). Additive manufacturing of specific ankle-foot orthoses for persons after stroke: A preliminary study based on gait analysis data. Math. Biosci. Eng..

[5] Sakib-Uz-Zaman C., Khondoker M.A.H. (2023). Polymer-Based Additive Manufacturing for Orthotic and Prosthetic Devices: Industry Outlook in Canada. Polymers.

[6] Wojciechowski E., Chang A.Y., Balassone D., Ford J., Cheng T.L., Little D., Menezes M.P., Hogan S., Burns J. (2019). Feasibility of designing, manufacturing and delivering 3D printed ankle-foot orthoses: A systematic review. J. Foot Ankle Res..

[7] https://get3d.pl/2020/05/13/jak-drukowac-z-petg/.

[8] (2022). Additive Manufacturing—General Principles—Fundamentals and Vocabulary.

[9] Banaszewski J., Pabiszczak M., Pastusiak T., Buczkowska A., Kuczko W., Wichniarek R., Górski F. (2018). 3D printed models in mandibular reconstruction with bony free flaps. J. Mater. Sci. Mater. Med..

[10] Shilo D., Emodi O., Blanc O., Noy D., Rachmiel A. (2018). Printing the Future-Updates in 3D Printing for Surgical Applications. Rambam Maimonides Med. J..

[11] Evill J. Cortex. https://www.evilldesign.com/cortex.

[12] Gorski F., Wichniarek R., Kuczko W., Hamrol A. (2019). Selection of Fused Deposition Modeling Process Parameters Using Finite Element Analysis and Genetic Algorithms. J. Mult.-Valued Log. Soft Comput..

[13] Novakova-Marcincinova L., Novak-Marcincin J., Barna J., Torok J. Special materials used in FDM rapid prototyping technology ap-plication. Proceedings of the 2012 IEEE 16th International Conference on Intelligent Engineering Systems (INES).

[14] Stano G., Ovy S.M.A.I., Edwards J.R., Cianchetti M., Percoco G., Tadesse Y. (2023). One-shot additive manufacturing of robotic finger with embedded sensing and actuation. Int. J. Adv. Manuf. Technol..

[15] Sepulveda-Navarrete D.A., Gutierrez P.S., Lopes A., Rome J.I., Goyal V.K., Espalin D. (2023). Ultrasonically embedded wires in multi-material parts produced by hybrid additive manufacturing. Addit. Manuf..

[16] Wang T.M., Xi J.-T., Jin Y. (2007). A model research for prototype warp deformation in the FDM process. Int. J. Adv. Manuf. Technol..

[17] Pilipovi A., Raos P., Šercer M. (2009). Experimental analysis of properties of materials for rapid prototyping. Int. J. Adv. Manuf. Technol..

[18] (2012). Abaqus.

[19] Aguilar-Martín J.J., Yagüe-Fabra J.A., Martínez J., Diéguez J.L., Ares E., Pereira A., Pérez J.A. (2013). Comparative between FEM Models for FDM Parts and their Approach to a Real Mechanical Behaviour. Procedia Eng..

[20] Górski F., Kuczko W., Weiss W., Wichniarek R., Żukowska M. (2019). Prototyping of an Individualized Multi-Material Wrist Orthosis Using Fused Deposition Modelling. Adv. Sci. Technol. Res. J..

[21] Kuznetsov V., Solonin A., Tavitov A., Urzhumtsev O., Vakulik A. (2019). Increasing strength of FFF three-dimensional printed parts by influencing on temperature-related parameters of the process. Rapid Prototyp. J..

[22] Srilakshmi C., Rao G.S., Kumar J.S. (2021). A Review on Damage Modelling and Analysis of Composites. Mater. Sci. Eng..

[23] Cazon A., Kelly S., Paterson A.M., Bibb R.J., Campbell R.I. (2017). Analysis and comparison of wristsplint designs using the finite element method: Multi-material three-dimensionalprinting compared to typical existing practice with thermoplastics. Proc. Inst. Mech. Eng. Part H J. Eng. Med..

[24] Marques M.A., Mendes E., Ramos N.V., Pinto V.C., Vaz M.A. Finite-element analysis of ankle-foot orthosis to predict fracture conditions during gait. Proceedings of the 1st ICH Gaia-Porto.

[25] Chen R.K., Chen L., Tai B.L., Wang Y., Shih A.J., Wensman J. Additive Manufacturing of Personalized Ankle-Foot Orthosis. Proceedings of the Transactions of the North American Manufacturing Research Institution of SME (NAMRC42).

[26] Hendricks A., Nevin S., Wikoff C., Dougherty M., Orlita J., Noorani R. (2018). The Low-Cost Design and 3D Printing of Structural Knee Orthotics for Athletic Knee Injury Patients. Int. J. Biomed. Biol. Eng..

[27] Cerda-Avila S.N., Medellín-Castillo H.I., de Lange D.F. (2019). Analysis and Numerical Simulation of the Structural Performance of Fused Deposition Modeling Samples with Variable Infill Values. ASME J. Eng. Mater. Technol..

[28] Li J., Tanaka H. (2018). Rapid customization system for 3D-printedsplint using programmable modelling technique—A practical approach. 3D Print. Med..

[29] https://sklep.rosa3d.pl/product/filament-pet-g-standard-175-mm-blue-sky-08kg/.

[30] (2019). Plastics—Determination of Flexural Properties.

